# Bioinformatics Approaches to Predict Mutation Effects in the Binding Site of the Proangiogenic Molecule CD93

**DOI:** 10.3389/fbinf.2022.891553

**Published:** 2022-06-21

**Authors:** Vittoria Cicaloni, Malancha Karmakar, Luisa Frusciante, Francesco Pettini, Anna Visibelli, Maurizio Orlandini, Federico Galvagni, Maurizio Mongiat, Michael Silk, Federica Nardi, David Ascher, Annalisa Santucci, Ottavia Spiga

**Affiliations:** ^1^ Toscana Life Sciences Foundation, Siena, Italy; ^2^ Structural Biology and Bioinformatics, Department of Biochemistry, University of Melbourne, Parkville, VIC, Australia; ^3^ Systems and Computational Biology, Bio21 Institute, University of Melbourne, Parkville, VIC, Australia; ^4^ Computational Biology and Clinical Informatics, Baker Heart and Diabetes Institute, Melbourne, VIC, Australia; ^5^ Baker Department of Cardiometabolic Health, Melbourne Medical School, University of Melbourne, Parkville, VIC, Australia; ^6^ Department of Biotechnology, Chemistry and Pharmacy, University of Siena, Siena, Italy; ^7^ Department of Medical Biotechnologies, University of Siena, Siena, Italy; ^8^ Department of Research and Diagnosis, Division Molecular Oncology, Centro di Riferimento Oncologico di Aviano (CRO) IRCCS, Aviano, Italy

**Keywords:** bioinformatics, computation biology, angiogenesis, CD93, multimerin

## Abstract

The transmembrane glycoprotein CD93 has been identified as a potential new target to inhibit tumor angiogenesis. Recently, Multimerin-2 (MMRN2), a pan-endothelial extracellular matrix protein, has been identified as a ligand for CD93, but the interaction mechanism between these two proteins is yet to be studied. In this article, we aim to investigate the structural and functional effects of induced mutations on the binding domain of CD93 to MMRN2. Starting from experimental data, we assessed how specific mutations in the C-type lectin-like domain (CTLD) affect the binding interaction profile. We described a four-step workflow in order to predict the effects of variations on the inter-residue interaction network at the PPI, based on evolutionary information, complex network metrics, and energetic affinity. We showed that the application of computational approaches, combined with experimental data, allowed us to gain more in-depth molecular insights into the CD93–MMRN2 interaction, offering a platform for developing innovative therapeutics able to target these molecules and block their interaction. This comprehensive molecular insight might prove useful in drug design in cancer therapy.

## Introduction

CD93 is a single-pass transmembrane glycoprotein belonging to the group XIV family of the C-type lectin-like domain (CTLD) superfamily (Zelensky and Gready, 2005), and it is predominantly expressed in endothelial cells (ECs) with expression also observed in monocytes, natural killer cells, platelets, myeloid cells, hematopoietic stem cells, and several lymphocyte subtypes (Greenlee et al., 2008; Khan et al., 2017). Notably, CD93 is highly expressed in blood vessels within tumors and has been identified as a key regulator of glioma angiogenesis (Langenkamp et al., 2015; Galvagni et al., 2017; Tosi et al., 2017), making it a suitable potential target for anti-angiogenic treatment. Recently, we identified a new signaling pathway involved in regulating EC adhesion and migration (Galvagni et al., 2016), but much remains to be clarified about the role of CD93 in the control of EC physiology. Recently, the pan-endothelial extracellular matrix (ECM) protein Multimerin-2 (MMRN2) was identified as the interacting partner of CD93 (Galvagni et al., 2017; Khan et al., 2017). We observed the CD93/MMRN2 interaction to be highly specific since no interaction was seen with other ECM molecules which share similar molecular domains with MMRN2 (Galvagni et al., 2017).

CD93 and MMRN2 are both up-regulated in tumor vasculature during tumor progression, suggesting that the CD93–MMRN2 interaction regulates tumor angiogenesis. Indeed, disruption of this interaction strongly impaired EC migration and *in vitro* angiogenesis (Galvagni et al., 2017). Recent work has suggested that inhibition of CD93–MMRN2 interaction may lead to disruption of vascular integrity in tumors, showing that the CD93–MMRN2 complex is required for the activation of β1 integrin, phosphorylation of focal adhesion kinase, and fibronectin fibrillogenesis in ECs (Lugano et al., 2018).

In the work of Galvagni et al. (2017), model structures and docking hypothetical studies about CD93–MMRN2 interaction were already performed. However, the interaction mechanism between these two proteins is yet to be studied. CD93–MMRN2 binding is dependent on a long-loop region in the C-type lectin-like domain (CTLD) of CD93, and this interaction is abrogated by point mutations in the CTLD and sushi-like domains (Khan et al., 2017; Galvagni et al., 2017). (For further information about CD93 and MMRN2 domains, see Supplementary Data Sheet Section S1. “Domains description.”) Here, the application of computational approaches, combined with experimental data [see Figure 7 of Galvagni et al., 2017 (Galvagni et al., 2017)], allowed us to gain more in-depth molecular insights into the CD93–MMRN2 interaction, working also to establish an *in silico* workflow model for shedding light on other unknown interacting molecules validated by experimental data.

## Methods

In this work, we compare experimental and *in silico* data to select the most matching docking pose.

Experimental data were handled as described in the Galvagni et al. (2017) article by using chimeric constructs containing the extracellular domains of CD93 fused to Myc, and the MMRN2 wild type fused to a His tag were generated as previously described (Orlandini et al., 2014; Colladel et al., 2016). Figure 7 of Galvagni et al. (2017) lists the mutations that contributed to the CD93–MMRN2 interaction and was the starting point for our bioinformatics investigation.

**FIGURE 1:**
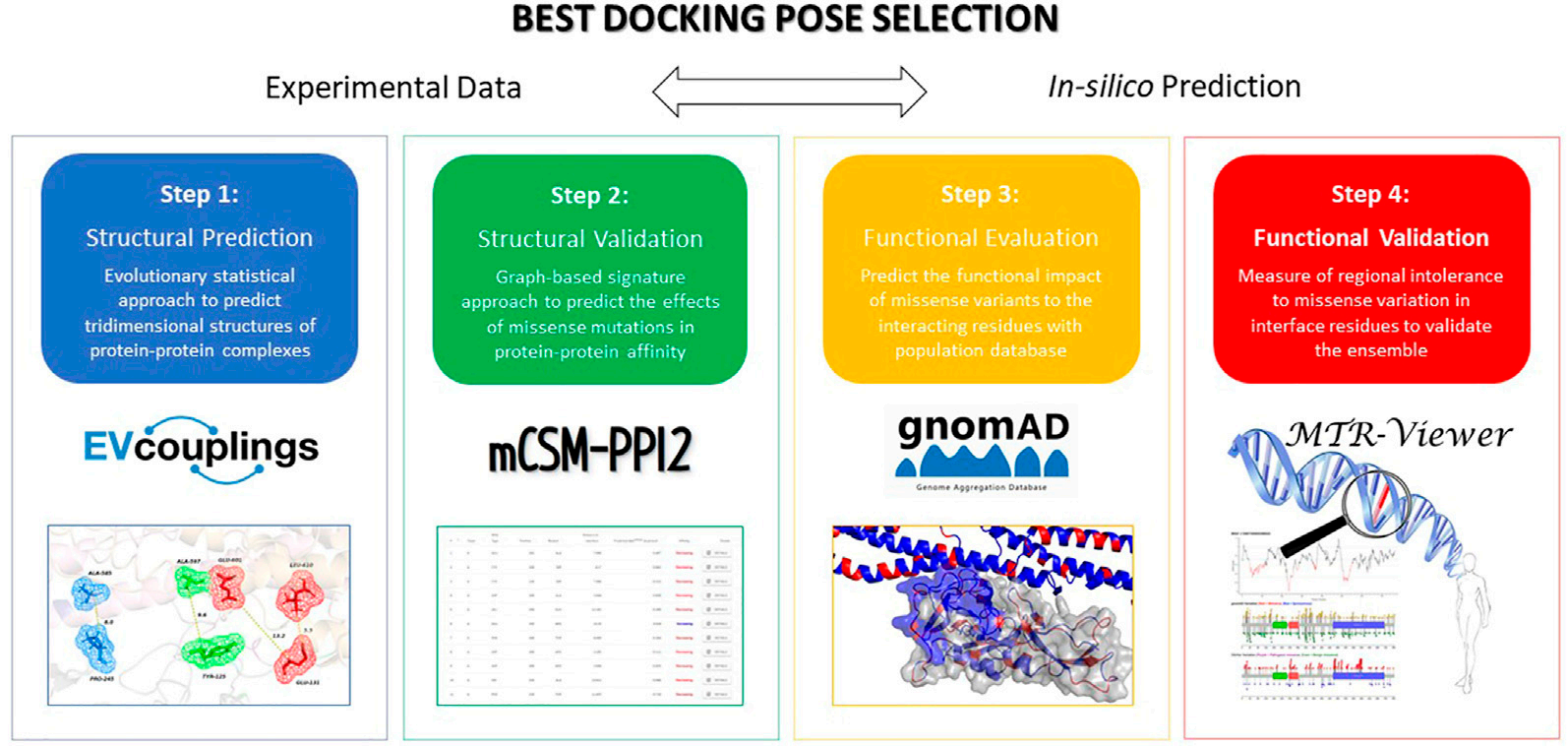
Four-step workflow applied to predict and evaluate the effects of variations on the inter-residue interaction network at the PPI.

In the same work, *in silico* model structures and docking studies were performed to investigate the region of CD93 and MMRN2 involved in the interaction (Galvagni et al., 2017). Starting from two docking poses, we applied a four-step workflow for screening the most trustworthy one (Figure 1).

We used the EVolutionary Couplings server to provide functional and structural information about proteins derived from the evolutionary sequence record, using methods from statistical physics (Hopf et al., 2019). By using FASTA sequence (UniProt code: Q9NPY3 and Q9H8L6 for CD93 and MMRN2, respectively), EVolutionary Couplings was used to determine co-evolved residues in our selected protein–protein interactions (PPIs) and to provide information on whether a protein interaction is conserved across enough sequenced genomes using a single pair per genome (Hopf et al., 2019).

We performed an *in silico* validation of the effect of CD93 mutants (Galvagni et al., 2017) by mCSM-PPI2 (Rodrigues et al., 2019). Using the transcripts ENST00000246006.5 and ENST00000372027 for CD93 (Chr20) MMRN2 (Chr10), respectively, we then mapped the gnomAD missense variants to the structure (Karczewski et al., 2021). The PPI interface of the complex was analyzed using the PDBePISA tool (Krissinel and Henrick, 2007). We calculated the measure of regional intolerance to missense variation for CD93 in both docked poses by using MTR (Galvagni et al., 2017; Traynelis et al., 2017; Silk et al., 2019).

## Results and Discussion

The aim of the PPI docking procedure is to predict correct poses and to score them according to the strength of interaction in a reasonable time frame. In this study, we presented an extended approach to evaluate the reliability of protein–protein complex structures, confirmed by experimental data. Starting from the best two plausible docking poses selected from the study by Galvagni et al. (2017), we applied a four-step workflow for screening the most trustworthy one. Based on an evolutionary statistical approach, our aim was to find co-evolved residues between CD93 and MMRN2: if a protein–protein interaction is conserved across enough sequenced genomes, using a single pair per genome can give accurate predictions of the interacting residues.

**FIGURE 2:**
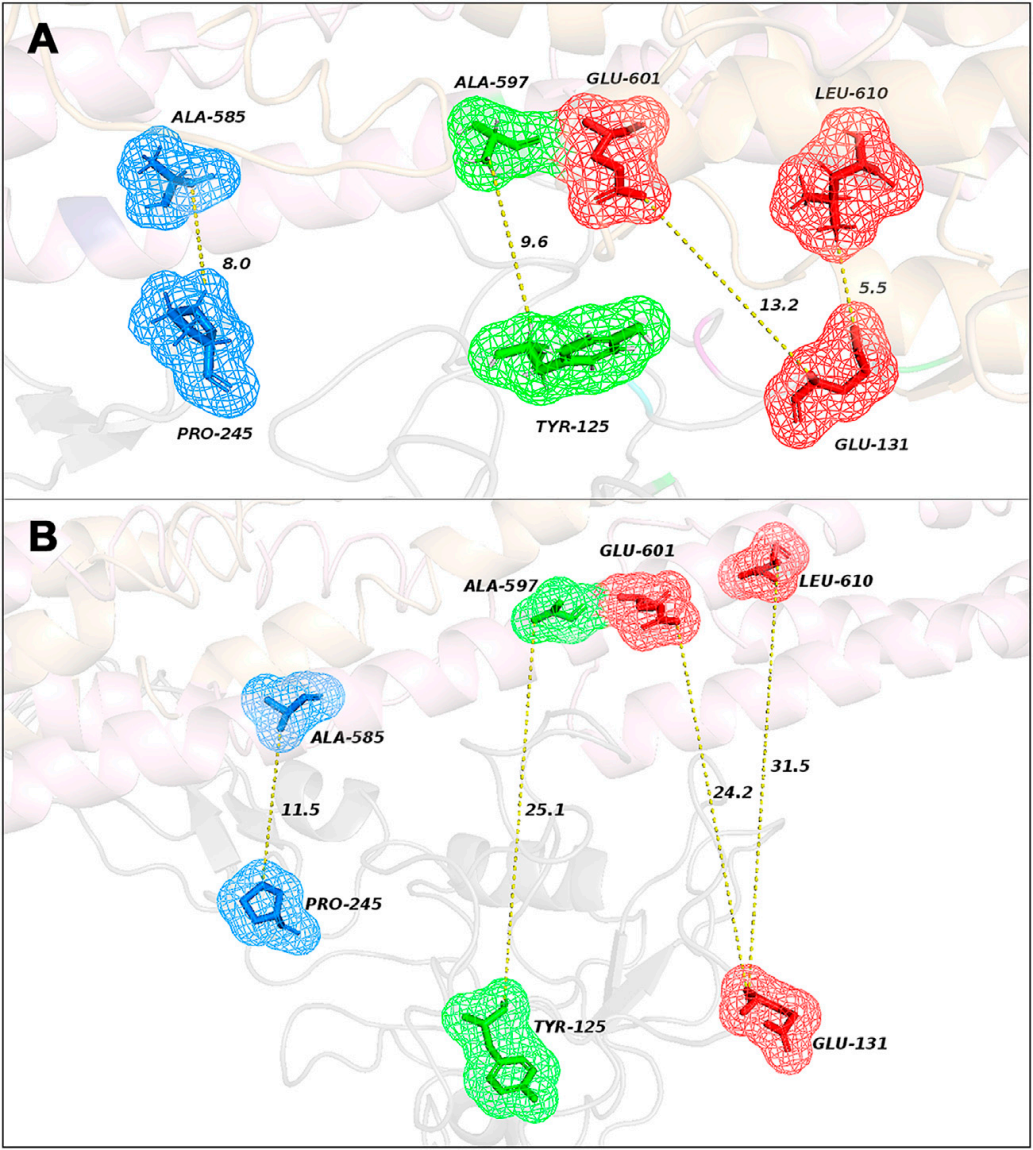
Representation of the closest co-evolved residue couples (colored sticks) in Pose 1 **(A)** and in Pose 2 **(B)** with their relative distances, resulting from EVcouplings analysis.

The analysis of correlated evolutionary sequence changes across proteins can identify residues that are in close proximity (below a threshold distance of 8 Å) with enough accuracy to determine the three-dimensional structure of the protein complexes; consequently, it can be used to screen the reliability of the selected poses (Hopf et al., 2014). All results are summarized in Table 1 and represented in Figure 2; moreover, a comprehensive output of such analysis is reported in “Supplementary Data Sheet Section S2.” Moreover, in “Supplementary Data Sheet Section S3,” the obtained results have been compared by using ColabFold (Mirdita et al., 2021). For Pose 1 (Figure 2A), the most interesting co-evolved residue couples are closer or equal to 8 Å: Leu-610 (in MMRN2) and Glu-131 (in CD93) with 5.5 Å and a high probability score of 0.82; Ala-585 (in MMRN2) and Pro-245 (in CD93) with 8 Å and a probability score of 0.70. However, the other two pairs (Ala-597 in MMRN2 and Tyr-125 in CD93; Ala-585 in MMRN2 and Pro-245 in CD93) showed an excellent probability score, respectively, of 0.98 and 0.70, and the distances are higher than the threshold of 8 Å. On the contrary, as shown in Figure 2B, there are no pairs of residues in Pose 2 with a distance below 8 Å. The co-evolved couple in the closest proximity is MMRN2 Ala-585/CD93 Pro-245 with a probability score equal to 0.70 and 11.5 Å. Differently from Pose 1, all the other selected pairs of Pose 2 presented distances larger than 20 Å. Being able to verify the presence of two co-evolved couples of residues in Pose 1 located at a reasonable distance, this first analysis suggested it as a probable more reliable pose.

**TABLE 1:** Summary of Evolutionary couplings results. Probability score and distance related to Pose 1 and Pose 2.

CD93	MMRN2	Probability	Distance (Å) Pose 1	Distance (Å) Pose 2
E131	E601	0.98	13,2	24,2
E131	L610	0.82	5,5	31,5
Y125	A597	0.71	9,6	25,1
P245	A585	0.70	8,0	11,5

The second step evaluated if the two selected poses fitted with experimental results derived from the study of Galvagni et al. (2017), where an extensive mutation analysis of the CD93/MMRN2 binding strength among wild-type and mutated proteins was evaluated by solid phase assay (see Table 2 for the full list of results). To characterize the interacting surface of CD93, point mutations were introduced, and the CD93 mutants expressed in 293T cells were analyzed using Western blots to assess the expression of the soluble recombinant proteins. Every experimental missense mutation was evaluated with mCSM-PPI2, a novel machine learning computational tool designed to predict the effects of missense mutations more accurately on protein–protein interaction binding affinity (Rodrigues et al., 2019). The predicted binding affinity scores were compared with experimental results: the poses that best fit with experimental results through the affinity prediction made with mCSM-PPI2 could be considered more reliable than the other one. For Pose 1 (Table 2), every binding strength prediction fitted with the experimental results apart from D249A (experimental results: increasing, prediction affinity: moderately decreasing) and H236A (experimental results: decreasing, prediction affinity: weakly increasing and with a distance to interface extremely elevated around 10 Å). For Pose 2 (Table 2), a major part of predictions fitted with experimental results with two serious exceptions for E100R (experimental results: slightly increasing, prediction affinity: strongly decreasing and with a distance to interface extremely close around 3.2 Å) and D249A (experimental results: increasing, prediction affinity: strongly decreasing, with a distance to interface of 3.2). In conclusion to this second step, Pose 1 fit the best with the experimental results.

**TABLE 2:** Binding affinity predictions. Every missense mutation of the two poses was evaluated with mCSM-PPI2; results were compared with experimental outcomes.

Mutated residue	Experimental data binding	Distance to interface		mCSM-PPI2 prediction		Affinity		Matching experimental vs. bioinformatics	
		Pose 1	Pose 2	Pose 1	Pose 2	Pose 1	Pose 2	Pose 1	Pose 2
E100R	Increase	6,571	3,215	0,131	−1,225	Increase	Decrease	Y	N
E242A	Decrease	5,402	4,288	−0,45	−0,646	Decrease	Decrease	Y	Y
D249A	Increase	5,084	3,259	−0,6	−1,311	Decrease	Decrease	N	N
L256Q	Decrease	1,67	2,403	−0,003	−0,035	Decrease	Decrease	Y	Y
F238T	Decrease	9,45	7,537	−0,804	−0,714	Decrease	Decrease	Y	Y
C104S	Decrease	6,855	5,392	−0,39	−0,26	Decrease	Decrease	Y	Y
D249R	Decrease	5,084	3,259	−0,405	−0,738	Decrease	Decrease	Y	Y
F248T	Decrease	7,269	7,23	−0,366	−0,236	Decrease	Decrease	Y	Y
C136S	Decrease	6,305	6,262	−0,335	−0,32	Decrease	Decrease	Y	Y
H236A	Decrease	9,904	9,649	0,028	−0,063	Increase	Decrease	N	Y

As a third step, we mapped the gnomAD missense variants to the structure docking poses (Karczewski et al., 2021), obtaining a b-factor equal to 1 or −1, where “1” indicates one or more missense variants found at this amino acid position and “−1” represents no missense variants seen at this amino acid position. Based on these results, we verified whether such residues are located at the binding interface. Thus, the complex structure of every pose was analyzed using the PDBePISA tool for the selection of interface residues (Krissinel and Henrick, 2007). By comparing gnomAD and PDBePISA, we noticed that in Pose 1, the total number of variants (excluding missense) seen at the interface positions of CD93 is larger than that in Pose 2. Specifically, in Pose 1, for a total of 47 interface residues, we observed 26 amino-acid variants’ positions, while in Pose 2, for a total of 36 interface residues, no missense variants were found in only 15 positions (Table 3). This finding suggests a greater tolerance for missense mutations in Pose 1 than in Pose 2, as confirmed by the following fourth step. The fourth step was based on the exploration of regional intolerance to missense variation in interface residues located in Pose 1 and Pose 2 by looking into missense tolerance ratio (MTR) (Traynelis et al., 2017; Silk et al., 2019) scores. It was crucial in this last step to evaluate missense variant deleteriousness by examining its surrounding regional intolerance and to calculate the MTR scores at their position (Table 3). There are no missense intolerant regions placed in the CD93 interfaces both in Pose 1 and in Pose 2. Summarizing our results, Pose 1 is the most plausible docking pose, consistent with experimental data.

**TABLE 3:** Results from mapping the gnomAD missense variants to CD93: b-factor = 1/−1, where “1” indicates one or more missense variants found at this amino acid position, and “−1” represents no missense variants seen at this amino acid position; next to each one is the associated MTR score.

Pose 1			Pose 2		
Residue	gnomAD	MTR	Residue	gnomAD	MTR
K64	−1	1	Q98	1	1
K103	−1	0,96	R99	−1	1
D106	−1	0,84	E100	1	1
P107	1	0,83	K101	1	0,97
S108	−1	0,86	K103	−1	0,96
L109	1	0,85	D106	−1	0,84
P110	−1	0,82	P107	1	0,83
L111	−1	0,84	S108	−1	0,86
K112	1	0,88	L109	1	0,85
W116	−1	0,82	P110	−1	0,82
G120	−1	0,78	G118	−1	0,76
E121	−1	0,76	G119	1	0,82
D122	1	0,78	E121	−1	0,76
T123	1	0,76	I137	1	0,8
P124	−1	0,77	S138	1	0,77
Y125	−1	0,8	K139	−1	0,74
S126	1	0,8	R140	1	0,73
N127	−1	0,83	G166	1	0,76
W128	−1	0,82	S167	−1	0,85
H129	−1	0,82	L191	1	1
K130	−1	0,81	A192	−1	1,01
E131	−1	0,81	L193	1	1
L132	−1	0,85	G194	1	1,03
R133	1	0,85	G195	1	1,02
N134	1	0,82	P196	1	0,97
S135	1	0,82	E213	−1	0,74
S138	1	0,77	K241	1	0,85
R140	1	0,73	E242	1	0,84
K159	−1	0,74	K243	1	0,85
E162	−1	0,76	D249	1	0,81
P164	1	0,74	W250	−1	0,82
A192	−1	1,01	G251	1	0,81
L193	1	1	S252	−1	0,82
G194	1	1,03	S253	1	0,82
G195	1	1,02	L256	−1	0,79
P196	1	0,97	L257	−1	0,77
K241	1	0,85			
K243	1	0,85			
A244	−1	0,84			
P245	−1	0,85			
V247	−1	0,86			
D249	1	0,81			
W250	−1	0,82			
G251	1	0,81			
S253	1	0,82			
L256	−1	0,79			
C257	−1	0,77			

## Conclusion

*In silico* and experimental procedures were used as a basis to determine the CD93 structure–function relationship. The CD93/MMRN2 complex was analyzed *in vitro*, dissecting interactions occurring in specific conditions. Protein docking procedures were used to predict the involvement of specific amino acid residues in the CD93/MMRN2 interaction. Structural analyses were conducted using bioinformatic tools for protein–protein interface regions, showing the key role of amino acid residues in the interaction. Here, we provide an approach to evaluate the best pose of protein–protein complex structures according to new experimental data. With the application of bioinformatic tools, we have described a four-step workflow to predict effects of variations on the inter-residue interaction network at the PPI, based on evolutionary information, complex network metrics, and energetic affinity; in addition, it allows us to map and explore regional intolerance to missense variation. These observations could provide a basis to strengthen the bioinformatics process involved in the development of new drugs.

## Limitation of This Study

A comparison of *in silico* and experimental procedures was effective for the determination of the structure–function relationship in a protein–protein interaction. However, it is important to emphasize that this exploratory study is based on only two docking poses; thus, it could be interesting to expand and further verify this four-step method on a larger case series to screen a higher number of potential docking poses (Orlandini et al., 2008).

## Data Availability

Publicly available datasets were analyzed in this study. These data can be found here: https://www.uniprot.org/uniprot, Q9NPY3, Q9H8L6.
