# Supplementary material for: Bioinformatics Approaches to Predict Mutation Effects in the Binding Site of the Proangiogenic Molecule CD93
Source: Front Bioinform. 2022 Jun 21;2:891553. doi: 10.3389/fbinf.2022.891553 (PMC9638713; doi:10.3389/fbinf.2022.891553)
Supplement: Supplementary file 1 [file DataSheet1.PDF]

Supplementary Materials
Section 1: Domains description

Description: Multimerin-2 (MMRN2)
Uniprot Accession: Q9H8L6
Source organism: Homo sapiens (Human)
Length: 949 amino acids
Pfam domains:

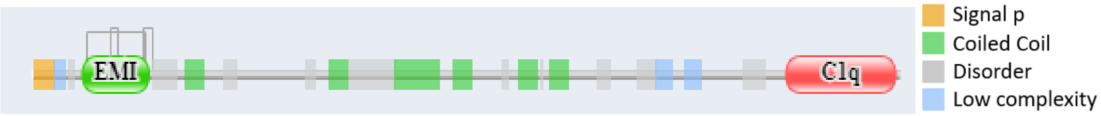

The **EMI domain**, first named after its presence in proteins of the EMILIN family, is a small cysteine-rich protein domain of around 75 amino acids. The EMI domain possesses six highly conserved cysteine residues, which likely form disulphide bonds. It has been suggested that the EMI domain could be a protein-protein interaction module as the EMI domain of EMILIN-1 was found to interact with the **C1q domain** of EMILIN-2. (http://pfam.xfam.org/protein/Q9H8L6).

Source	Domain	Start	End
sig_p	n/a	1	22
low_complexity	n/a	3	19
low_complexity	n/a	22	35
disorder	n/a	40	46
Pfam	EMI	55	127
disorder	n/a	132	158
coiled_coil	n/a	167	187
low_complexity	n/a	169	178
disorder	n/a	176	186
disorder	n/a	210	218
disorder	n/a	220	224
disorder	n/a	300	310
coiled_coil	n/a	326	346
disorder	n/a	337	376
disorder	n/a	380	397
coiled_coil	n/a	398	446
coiled_coil	n/a	461	481
low_complexity	n/a	467	479
disorder	n/a	515	516
disorder	n/a	520	521
coiled_coil	n/a	533	553
disorder	n/a	558	559
coiled_coil	n/a	568	588
low_complexity	n/a	577	585
disorder	n/a	620	622
disorder	n/a	624	627
disorder	n/a	629	633
disorder	n/a	663	692
low_complexity	n/a	684	701
low_complexity	n/a	716	734
disorder	n/a	780	803
Pfam	C1q	827	946
disorder	n/a	889	892

Table 1. In this table it is possible to notice the two domains of MMNR2 (EMI and C1q), the coiled coil, the disorder regions, and the low complexity regions.

Description: Complement component C1q receptor (CD93)
Uniprot Accession: Q9NPY3
Source organism: Homo sapiens (Human)
Length: 652 amino acids
Pfam domains:

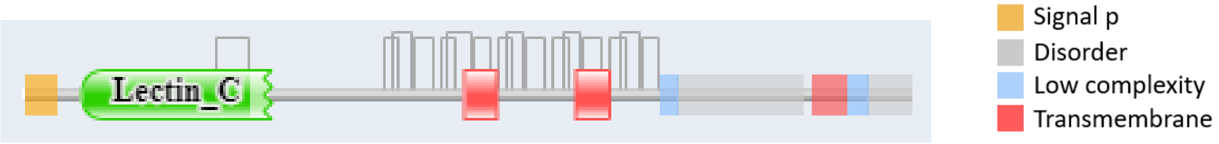

A C-type lectin (CLEC) is a carbohydrate-binding protein domain better known as lectin. Generally, proteins that contain C-type lectin domains have a diverse range of functions including cell-cell adhesion, immune response to pathogens and apoptosis (http://pfam.xfam.org/family/PF00059). The EGF-like domain is an evolutionary conserved protein domain, which derives its name from the epidermal growth factor. It is very common and includes about 30 to 40 amino acid residues (http://pfam.xfam.org/family/PF12662).

Source	Domain	Start	End
sig_p	n/a	1	23
low_complexity	n/a	7	15
Pfam	Lectin_C	42	181
disorder	n/a	57	60
low_complexity	n/a	143	158
low_complexity	n/a	161	173
disorder	n/a	163	166
Pfam	cEGF	325	348
Pfam	cEGF	406	430
disorder	n/a	469	574
low_complexity	n/a	470	481
transmembrane	n/a	582	606
low_complexity	n/a	586	605
low_complexity	n/a	607	620
disorder	n/a	610	632
disorder	n/a	635	652

Table 2. In this table it is possible to notice the three domains of CD93 (CLEC and two cEGFs), the disorder regions, and the low complexity regions.

The interaction region for CD93 is represented by C-type lectin domain (CLEC), whereas for MMRN2 the residues involved in the interaction are placed in a coiled coil region.

Supplementary file

Section 3

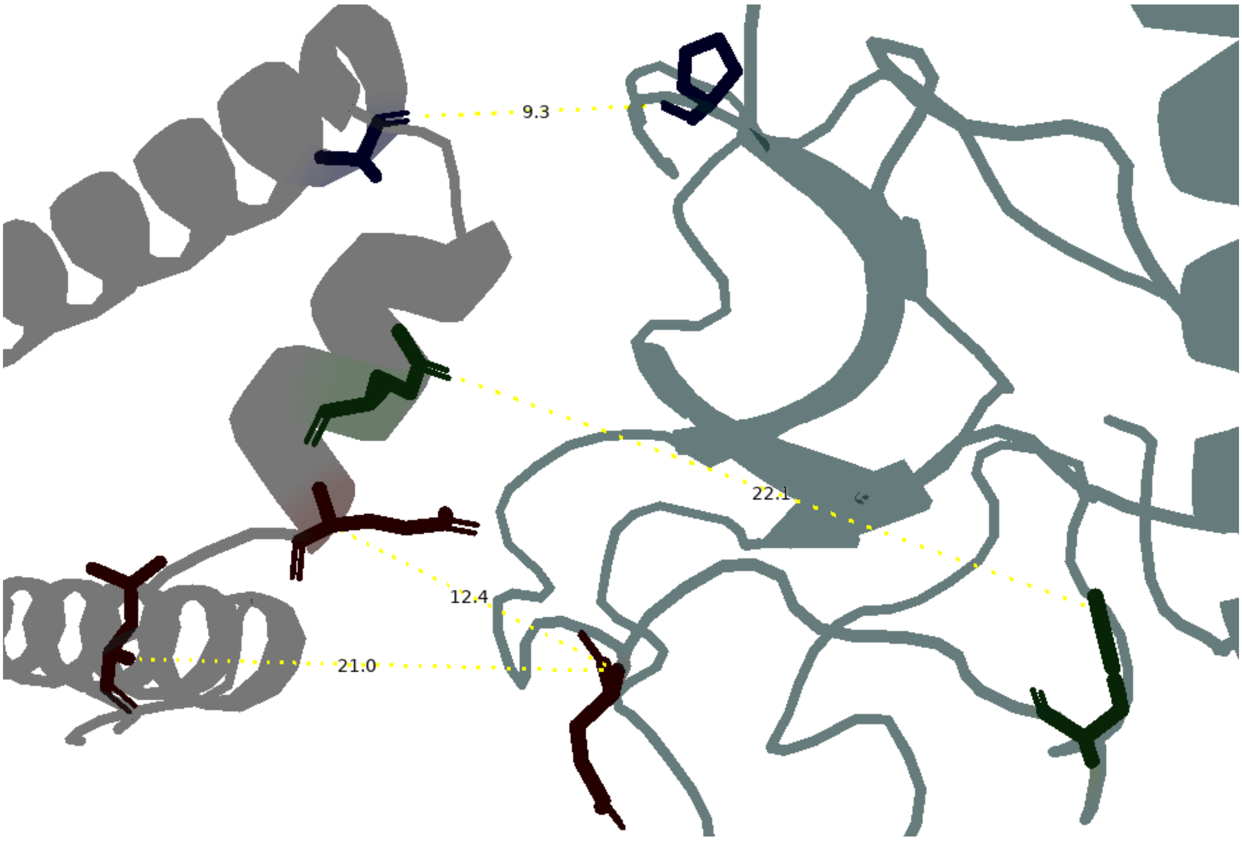

Fig. 1. Structure prediction model powered by AlphaFold2 and RoseTTAFold by using ColabFold.

CD93	MMRN2	Probability	Distance (Å) POSE 1	Distance (Å) POSE 2	Distance (Å) By ColabFold
**E131**	**E601**	0,98	13,2	24,2	12,4
**E131**	**L610**	0,82	5,5	31,5	21,0
**Y125**	**A597**	0,71	9,6	25,1	22,1
**P245**	**A585**	0,70	8,0	11,5	9,3

Table 1. Evolutionary coupling results summary. Probability score and distance related to Pose 1 and related to Pose 2 and distances obtained by ColabFold prediction model.
